# Prognostic Models for Disease Progression and Outcomes in Chronic Obstructive Pulmonary Disease: A Systematic Review and Meta-Analysis

**DOI:** 10.3390/jcm14248725

**Published:** 2025-12-09

**Authors:** Deborah Testa, Pietro Magnoni, Caterina Fanizza, Martino Bussa, Adele Zanfino, Dariush Khaleghi Hashemian, Paola Rebora, Lucia Bisceglia, Antonio Giampiero Russo

**Affiliations:** 1 Unità di Epidemiologia, Agenzia di Tutela della Salute (ATS) della Città Metropolitana di Milano, Via Conca del Naviglio 45, 20123 Milan, Italy; 2 Area Epidemiologia e Care Intelligence, Agenzia Regionale Strategica per la Salute ed il Sociale (AReSS) Puglia, 70121 Bari, Italy; 3 Centro Interdipartimentale Bicocca Bioinformatics Biostatistics and Bioimaging Centre (B4), Dipartimento di Medicina e Chirurgia, Università Milano Bicocca, 20126 Vedano al Lambro, Italy; 4 Biostatistics and Clinical Epidemiology, Fondazione IRCCS San Gerardo dei Tintori, 20900 Monza, Italy

**Keywords:** systematic review, meta-analysis, chronic obstructive pulmonary disease, prognostic models, machine learning, risk stratification

## Abstract

**Background/Objectives**: Prevalence and burden of chronic obstructive pulmonary disease (COPD) are projected to increase in the coming decades. Although prognostic models for disease progression and exacerbation risk have proliferated, especially with the advent of machine learning (ML), their methodological rigor, generalizability, and predictive performance remain inconsistent. This study aimed to systematically review prognostic models for disease progression in adults with COPD, including traditional regression-based methods and ML techniques, evaluating model performance, sources of heterogeneity and methodological issues. **Methods**: PubMed and Embase were searched for all studies that developed and/or validated prognostic models for mortality (overall and cause-specific), exacerbations, or hospitalizations in adults with COPD over a time window of 1–5 years. Methodological quality was appraised using PROBAST. Model performance was summarized descriptively, and discrimination (c-statistic) was meta-analyzed for externally validated models with sufficient homogeneity. **Results**: Eighty-seven studies presenting 193 prognostic models across 96 unique cohorts were included. Only 7% of models were based on ML. Thirty-eight percent of records were validations of multidimensional indices. All-cause mortality (n = 85), severe exacerbations (n = 38) and moderate/severe exacerbations (n = 16) were the most frequently studied outcomes. Meta-analysis of exacerbation models was hampered by insufficient homogeneity (median c 0.74). As for mortality, BODE outperformed other indices (pooled c 0.75). Over 40% of studies were flawed by a high risk of bias. **Conclusions**: Despite a comprehensive literature search and thorough data extraction, we were able to provide a meaningful quantitative synthesis only for externally validated mortality models, as pooling results for other individual outcomes was precluded by substantial heterogeneity. Our findings highlight the predominance of regression approaches, the limited use of ML, the presence of persistent methodological limitations and the need for more robust, validated models capable of handling complex, multimodal patient data.

## 1. Introduction

Chronic obstructive pulmonary disease (COPD) has been defined by the Global Initiative for Chronic Obstructive Lung Disease (GOLD) as “a heterogeneous lung condition characterized by chronic respiratory symptoms (dyspnea, cough, sputum production and/or exacerbations) due to abnormalities of the airways (bronchitis, bronchiolitis) and/or alveoli (emphysema) that cause persistent, often progressive, airflow obstruction” in 2023 [1]. According to this definition, its estimated worldwide prevalence was approximately 10% in 2022, corresponding to 480 million cases [2]. The prevalence of COPD is nearly twice as high in males as in females worldwide but is almost equal between sexes in developed countries. It increases with age, being five times higher in individuals over 65 compared to those under 40 [1,3]. COPD is also more frequent in smokers and in individuals of lower socioeconomic status [3,4]. It is a major cause of mortality, ranking as the eighth leading cause of death worldwide and the third among non-communicable diseases in 2021 [5]. Its global macroeconomic burden for the period 2020–2050 has been estimated at 4326 trillion 2017INT$, with the majority attributed to the USA, China, India and a few European Countries [6]. The 2023 GOLD report [1] and the work of Boers and colleagues [2] indicate that the prevalence and impact of COPD are expected to rise in the coming decades, due to the combination of global population aging and continued exposure to risk factors associated with COPD [2,7,8]. In fact, COPD arises from a combination of genetic predisposition and environmental factors interacting throughout an individual’s life. The most influential known risk factors are active or passive cigarette smoking, biomass and pollution exposure, asthma, airways infections, abnormal lung development, and genetic factors [1,9]. Comorbidities have a significant impact on COPD by influencing its clinical manifestation, complicating its management, and increasing both overall severity and mortality risk. Approximately two-thirds of patients diagnosed with COPD have one or two comorbidities [9], most frequently including ischemic heart disease, heart failure, pulmonary hypertension, and diabetes [10].

In addition to being a progressive disease, COPD is characterized by acute exacerbations that, if severe, may lead to hospitalization and the need for ventilatory support. The GOLD classification divides COPD into four categories, based on patient-reported symptoms and the frequency of acute exacerbations. It also classifies airflow limitation into four stages based on spirometry results, particularly the forced expiratory volume in one second (FEV_1_) [1]. Various models have been developed for predicting COPD progression (worsening symptoms, FEV_1_ decline, or the need for non-invasive ventilatory support), acute exacerbations and their consequences (hospitalization, admission to an intensive care unit, invasive ventilation), and mortality [11,12,13,14]. They include multivariable regression models, which are the most commonly reported in the literature, as well as more recent applications of machine learning (ML). They utilize health administrative databases and/or specific medical datasets, encompassing a wide range of demographic, socio-economic, and clinical prognostic factors. Several multidimensional indices have also been specifically developed for COPD patients to support clinical management. These indices combine multiple variables, capturing different aspects of this complex and heterogeneous disease. Each index has strengths and limitations in its prognostic capacity for COPD-related outcomes, depending on the clinical scenario [15].

A systematic review of the numerous models developed for medium- and long-term outcomes is warranted to identify which predictors and techniques yield the highest predictive accuracy. The few systematic reviews available on this subject to date have primarily focused on multivariable regression models, excluding ML approaches [11,12,13,14]. Furthermore, published systematic reviews often evaluate only a single outcome and have not been updated recently.

This systematic review aimed to update the current state of research with a novel, comprehensive synthesis of prognostic models for COPD progression in the adult population over a time window of 1–5 years, along with a thorough evaluation of their predictive performance. Quantitative assessment was conducted via meta-analysis of c-statistics whenever judged feasible, based on sufficient homogeneity of outcomes, predictors, and prediction intervals across models. When meta-analysis was not judged feasible, we provided a descriptive summary of model performance and an analysis of factors influencing it, including types of predictors and models, study setting, and sources of bias. Both traditional regression models and ML techniques were included to map their current use. By assessing methodological robustness in terms of risk of bias, this study also aims to highlight gaps in the existing literature regarding study quality and the reliability of prognostic models for COPD.

## 2. Materials and Methods

This systematic review was conducted following the standard guidelines provided by the CHecklist for critical Appraisal and data extraction for systematic Reviews of prediction Modelling Studies (CHARMS) [16], and reported in accordance with the Preferred Reporting Items for Systematic Reviews and Meta-Analyses (PRISMA) 2020 statement [17]; the completed PRISMA 2020 checklist is provided in Supplementary Table S1. The risk of bias and the applicability of the included models were assessed using the Prediction model Risk Of Bias Assessment Tool (PROBAST) [18]. The free web tool CADIMA was used to facilitate collaboration among reviewers, manage search results, track decisions, and document the study process [19]. The protocol for this systematic review and meta-analysis was registered and published in the International Prospective Register of Systematic Reviews (PROSPERO) with registration number CRD42023456687. A complete methodological workflow outlining all review steps, the logical flow of results, and the approach to data synthesis and presentation is provided in Supplementary Figure S1.

### 2.1. Literature Search

The search was performed on 08 August 2023 in both PubMed and Embase full-text archives. The search string used combinations of terms related to COPD, prediction/prognosis, modeling methods, and disease progression or exacerbations, and it was validated by an experienced librarian to ensure comprehensive coverage of articles on prognostic models for COPD progression published since 01 January 2012, in any language. The complete PubMed and Embase search strings are provided in Supplementary Table S2. The results obtained through this search strategy were manually integrated with references from the full-text included studies and previous systematic reviews. Abstracts and conference proceedings were also screened for inclusion.

### 2.2. Study Selection

The review question for this study was defined according to the PICOTS framework, as follows. “Population”: adult (≥18 years) human subjects with a diagnosis of COPD. “Index intervention/Exposure”: any developed, internally or externally validated, multivariable regression models and ML algorithms predicting clinical outcomes of COPD using demographic, socio-economic, and clinical prognostic factors; the prognostic/predictive factors under analysis could be derived from health administrative databases, clinical records, and medical data collected specifically for the study; studies validating multidimensional indices used to define prognostic scores (ADO, B-AE-D, BODE, BODEX, CODEX, DOSE) were also assessed [15]. Details of their construction are provided in Supplementary Table S3. “Comparator”: not applicable to this review. “Outcome”: prediction accuracy, evaluated through the c-statistic, of models predicting the risk of COPD progression (symptoms worsening, FEV_1_ decline, need for non-invasive ventilatory support), exacerbations and their sequelae (hospitalization, ICU admission, invasive ventilation), and all-cause or COPD-specific mortality. “Timing”: prediction window between 1 and 5 years. “Setting”: all predictive models intended for use by healthcare professionals at any point during the natural history of COPD; studies applying models to the general population or to patients recruited in outpatient or inpatient settings were included and assessed separately. A final eligibility criterion was added to exclude cross-sectional study designs, thereby restricting inclusion to studies with longitudinal designs appropriate for evaluating COPD prognostic models.

References resulting from the search strategy were imported into CADIMA and double-screened independently by at least two of three reviewers (DT, CF, MB) to identify eligible studies. All selection criteria were assessed independently during the screening process, both at the title/abstract level and then at the full-text level. Any disagreements were resolved through discussion until consensus was reached, or by a third reviewer.

### 2.3. Data Extraction

From each included study, both study-specific and model-specific data were extracted. Considering the broad scope of this research question, a single study could yield more than one eligible model if evaluating more than one outcome, performing validation on several cohorts, and/or specifying different model versions with varying sets of predictors. Retrieved data included: study identification and design (authors, title, year of publication, location and time of enrollment, setting, eligibility criteria), demographical and clinical characteristics of enrolled subjects (sample size, age and sex distribution, disease duration and stage, treatment, comorbidities), follow-up duration and patient outcomes (proportion of patients experiencing the event, proportion remaining at follow-up), model specification (modelling method, included predictors and their data sources, variable selection technique) and performance considering both discrimination and calibration (type of validation, c-statistic with 95% confidence interval at different time points between 1 and 5 years, observed/expected ratio, Brier score, calibration slope). The full list of collected variables can be found in the data extraction form, provided as Supplementary Table S4.

During data collection, we identified several limitations arising from inconsistencies in variable reporting across studies. To mitigate these issues, we applied a series of harmonization strategies. Specifically, we standardized variable entry and converted measurements into common reference units to improve comparability. When population-level statistics were reported only for subgroups rather than the full sample, we reconstructed overall estimates using weighted averages to ensure that summary measures accurately reflected the total study cohort. Additionally, when not explicitly reported by the original studies, we derived supplementary metrics, such as confidence intervals or standard errors, from the available raw data. All data were extracted independently by at least two of three reviewers (DT, CF, MB) and collected using a standardized form adapted from the CHARMS checklist. Several rounds of piloting were initially performed by the reviewers to refine the extraction fields. Any disagreements were resolved through discussion until consensus was reached, or by a third reviewer.

To summarize the number and type of prognostic factors included as final predictors, we categorized them into: sex; age; ethnicity; socioeconomic status (including variables related to education, occupation, deprivation index); alcohol use; smoking status; history of the present illness (HPI), which includes clinical variables describing COPD progression, associated manifestations/symptoms, severity and duration; past medical history (complications and comorbidities); family history; medical therapy (systemic and/or inhaled therapy); variables derived from physical examination and vital signs; laboratory tests (blood, sputum); spirometry results; imaging tests (i.e., computed tomography, CT). We also investigated the inclusion of common multidimensional indices used in COPD assessment, such as ADO, BODE and DOSE, their variants, as well as different versions of the GOLD ABCD grading system (2007/2011/2013/2017/2021).

### 2.4. Critical Appraisal

Each eligible study included in the systematic review was assessed to explore its applicability to the review question and the presence of bias using PROBAST, which is the tool for assessment of risk of bias (ROB) in prognostic model studies [18]. PROBAST provides a structured approach and comprises four domains, for a total of 20 signaling questions that aid in the assessment of ROB and applicability concerns. The “Participants” domain covers potential sources of bias and applicability issues related to the data source used, study design, and how the participants were selected. The “Predictors” and “Outcome” domains relate to how these were defined and measured, including aspects of standardization, reproducibility, and blinding. Finally, the “Analysis” domain assesses the correct handling of essential statistical factors such as sample size, model building, handling of missing data and class imbalance. The full list of items can be found in Supplementary Table S5, containing results of the evaluation for all included studies. Risk of bias and applicability were assessed separately for each domain. When a prediction model received low risk ratings in all domains, it was considered to have “low ROB”. If at least one domain received an unclear risk rating, the model was classified as having “unclear ROB”, and if at least one domain received a high risk rating, it was classified as having “high ROB”. Likewise, a model was considered to have “low applicability concern” if all domains were rated low, “unclear applicability concern” if at least one domain was unclear, and “high applicability concern” if at least one domain was rated high.

### 2.5. Meta-Analysis

Model performance, as measured by the discrimination metric (c-statistic), was summarized descriptively for the included studies. A meta-analysis was conducted whenever at least two externally validated models were homogeneous in terms of outcome, predictors, and prediction interval (1 year, 2–3 years, 4–5 years). To minimize potential bias [20], we also performed subgroup analyses by study setting (outpatient/primary care vs. inpatient) and including only studies judged at low ROB for key PROBAST items considered by the reviewers as likely to influence the discrimination metric and summary estimates (list in Supplementary Table S5). Meta-analysis was performed in R (version 4.5.2) using the valmeta() function from the metamisc package (version 0.4.0).

## 3. Results

**Figure 1:**
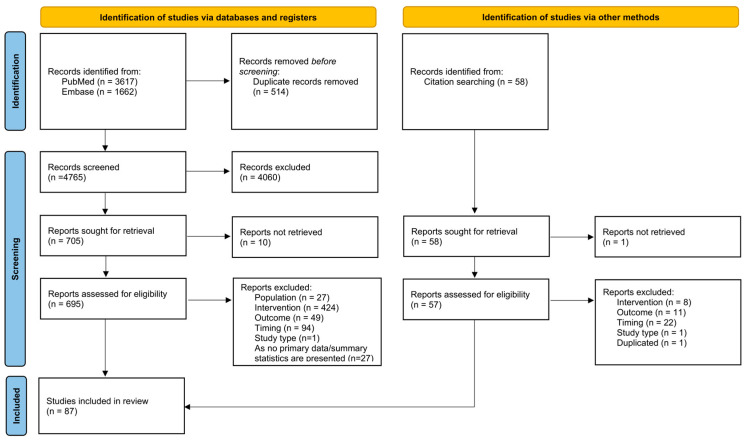
PRISMA flow diagram (source: Page et al., 2021 [17]).

Applying the research strings, 3617 studies were retrieved from PubMed and 1662 from Embase. After deduplication, 4765 articles were screened at the title/abstract level, and 4060 of these were excluded according to the defined eligibility criteria. The remaining 705 articles were assessed in full-text for eligibility, together with the 57 articles retrieved through citation searching. From this pool, 665 articles were excluded. Details on reasons for exclusions at each phase are reported in the PRISMA diagram (Figure 1). Finally, from the 87 studies (84% full papers, 16% abstracts) which met all the eligibility criteria, 193 individual prognostic models were extracted and included in our review [21,22,23,24,25,26,27,28,29,30,31,32,33,34,35,36,37,38,39,40,41,42,43,44,45,46,47,48,49,50,51,52,53,54,55,56,57,58,59,60,61,62,63,64,65,66,67,68,69,70,71,72,73,74,75,76,77,78,79,80,81,82,83,84,85,86,87,88,89,90,91,92,93,94,95,96,97,98,99,100,101,102,103,104,105,106,107].

We first analyzed general characteristics of the COPD cohorts enrolled in the extracted studies. Some studies contributed more than one distinct cohort. Multiple records corresponding to the same cohorts across different studies were merged, so that each cohort would only contribute once to the descriptive statistics. In all, 96 unique study cohorts were analyzed, encompassing a total of 1,438,040 patients. Of these, 95% were from purely observational studies and the remaining 5% were cohorts originally recruited for clinical trials. Patients were mostly recruited from outpatient/primary care settings (75 cohorts, 78.1%), whereas fewer studies were performed on hospitalized patients (15 cohorts, 15.6%). Three studies included patients recruited from a mix of the previous two categories, and three studies did not clearly specify the clinical setting of enrollment. The COPD cohorts considered across the studies were predominantly male (67.8%), with a mean age ranging from 58.8 to 76.3 years (median 68 years). Only a few studies (around 27%) reported information about COPD severity in terms of GOLD stage; patients with moderate or severe COPD (GOLD stages II–III) were more frequently represented compared to those with mild (I) or very severe (IV) disease. This pattern was also reflected in the mean FEV_1_% predicted (median 49.1, Q1–Q3 47.4–59.9). Information about the medical therapy (use of inhaled corticosteroids, long-acting β2-agonists, long-acting muscarinic antagonists) was described for very few cohorts (17.7–22.9%) and their reported prevalence of use was widely heterogeneous. Data on disease duration and comorbidities were reported for too few cohorts (6 and <10, respectively) to allow descriptive analyses providing meaningful insights. This highlights substantial gaps in reporting across primary studies with regard to crucial demographic, anthropometric, clinical, and therapy-related variables. Summary distributions of these variables, based on the information available, are provided in Table 1. The recruitment setting significantly impacted baseline characteristics: hospitalized patients tended to be older (72 vs. 67 years), had slightly higher smoking exposure (48.5 vs. 46.4 pack-years), and a greater proportion had severe disease (53.5% in GOLD stages III/IV vs. 42.7%) compared with the general population. However, these observations should be interpreted cautiously, as reporting completeness also varied considerably between the two settings (Supplementary Table S6).

**Figure 2:**
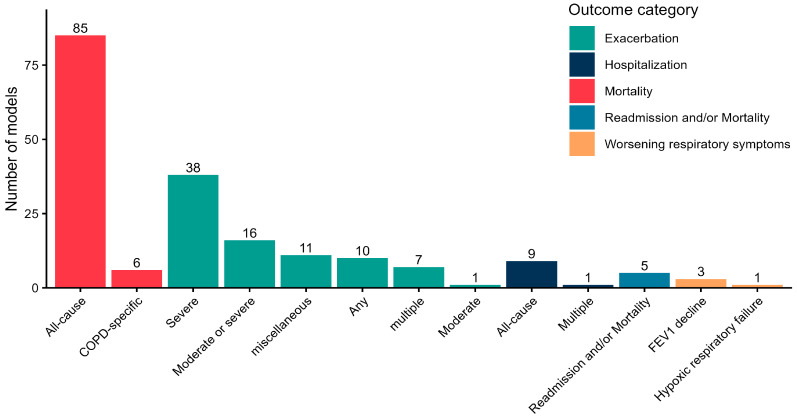
Bar chart of COPD-related outcomes predicted by the prognostic models (n = 193).

We then explored the clinical outcomes predicted by the 193 models (Figure 2). A preliminary classification into five broad categories showed that the most frequently investigated outcomes were mortality and exacerbations (which include COPD-specific hospitalizations), assessed by 47% (n = 91) and 40% (n = 78) of the prognostic models, respectively. All-cause hospitalizations (n = 10) and worsening of respiratory symptoms (n = 9) were less frequently represented. Five models assessed a composite outcome defined as hospital readmission and/or mortality. ICU admission and the need for invasive ventilation were not captured by the time window considered in this review. At a more detailed level, mortality was predominantly assessed as all-cause mortality (n = 85), rather than COPD-specific mortality (n = 6). According to the GOLD definitions, moderate exacerbations are those requiring short-acting bronchodilators plus antibiotics and/or oral corticosteroids, whereas severe exacerbations require hospitalization or an emergency room visit [1]. Stratifying exacerbations by severity, most models included only severe exacerbations (n = 38), followed by moderate or severe exacerbations (n = 16), and any exacerbations (n = 10). Seven models were developed for multiple exacerbations (more than one event within the study period required to adjudicate the patient as having the outcome), and eleven models used various composite definitions of exacerbation outcomes. We observed substantial variability arising from differences in the definition of the exacerbation outcome itself. In several studies, exacerbations of varying severity and frequency were combined into a single composite outcome, which hindered direct comparability across models.

**Figure 3:**
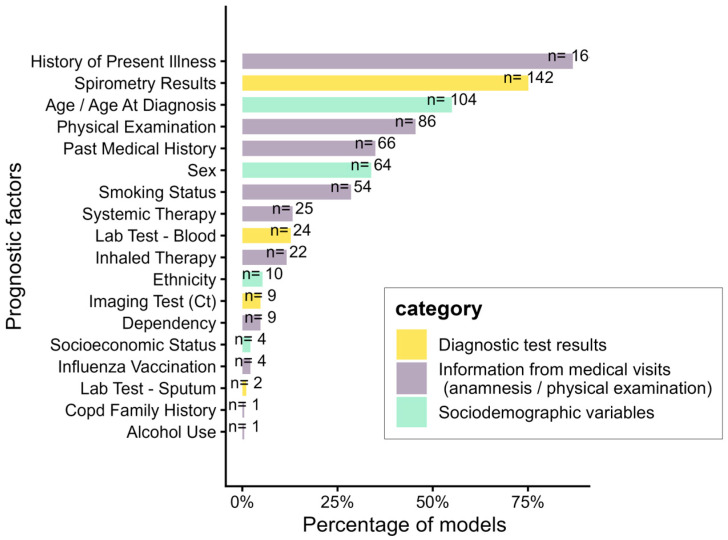
Prognostic factors usage in all extracted models (n = 193).

Apart from demographics, the predictor categories most frequently used across all models were HPI (86.2%), spirometry results (75.0%), physical examination (45.2%) and past medical history (35.1%). Laboratory tests and imaging results were included in a minority of models (13.8% and 4.8%, respectively). More details are found in Figure 3. 

About half of all models included multidimensional indices among the final predictors: 27.6% included one version of the ABCD GOLD stages, 23.4% included BODE or its modified version, and 18.1% included ADO or its modified versions. Other indices were used in fewer than 10% of the models.

Values of the c-statistic for the included models (with 95% confidence intervals) varied widely, ranging from 0.41 (0.38–0.45) to 0.93 (0.87–0.99) at 1 year, from 0.60 (0.52–0.69) to 0.91 (0.86–0.97) at 2–3 years, and from 0.59 (0.52–0.66) to 0.84 (0.80–0.87) at 4–5 years. Of note, only 17 models (8.8%) were accompanied by reported calibration metrics, including any of the observed/expected ratio, Brier score, or calibration slope. Full details of the extracted discrimination and calibration metrics are provided in Supplementary Table S4.

**Figure 4:**
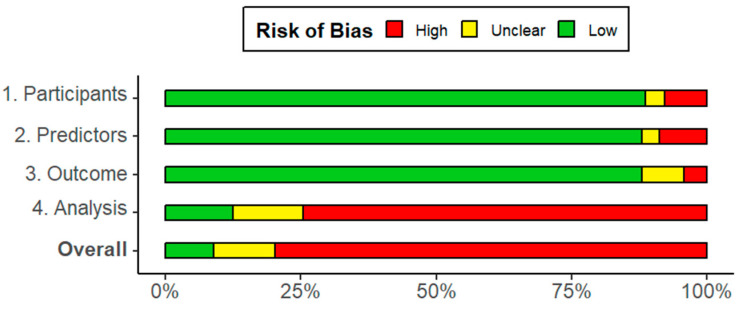
Risk of bias assessment (PROBAST) of all extracted models (n = 193).

Each extracted model was assessed for applicability and ROB using PROBAST (Supplementary Table S5). Considering our broad research question, there were few issues regarding applicability, as only 6.2% of models (most of which derived from abstracts/proceedings) received an overall judgement of unclear or high concern due to insufficient information. In contrast, 91.1% of the models were judged to have a high or unclear ROB, which raises important caveats for their potential clinical use and substantially limits the generalizability of their reported results (Figure 4).

A deeper analysis of these aspects, including model predictive performances and meta-analyses when feasible, is presented in the following sections for the three most represented specific outcomes: all-cause mortality, severe exacerbations, and moderate or severe exacerbations.

### 3.1. Overall Mortality

The prognostic models predicting overall mortality (n = 85) [21,22,23,24,25,26,27,28,29,30,31,32,33,34,35,36,37,38,39,40,41,42,43,44,45,46,47,48,49,50,51,52,53,54,55,56,57,58,59,60,61,62,63,64] were developed predominantly using traditional statistical techniques (96.5%), with Cox regression being the most frequently applied method (56.5%), followed by logistic regression (37.6%). Machine learning approaches were applied for only three models (3.5%), specifically using support vector machines (n = 2) and random forest algorithms (n = 1). At least half of the models reported estimates of model performance at 1 year (52.9%) and at 2 or 3 years (57.6%), while fewer models (23.5%) evaluated longer-term mortality (4 or 5 years). Most models were applied in outpatient/primary care settings (n = 67) as opposed to hospitalized patients (n = 13). The clinical setting of patient recruitment appears to be a key source of heterogeneity in the results, beginning with the observed frequency of the mortality event (6.8% in outpatients vs. 15.9% in hospitalized patients; findings from 61% of studies, where this calculation was feasible based on the availability of both sample size and reported number of events). This confirms that patients hospitalized for an acute exacerbation or other significant clinical events constitute a distinct “high-risk” subgroup compared to the general population, warranting recalibration of baseline risks when transferring models developed in one setting to another. The most frequently used predictors/categories were HPI and spirometry results (81.2% and 75.3%, respectively), followed by age (51.8%), physical examination (45.9%), past medical history (42.4%), sex (22.4%), smoking status (15.3%) and blood test results (9.4%). All other predictors were used in less than 3% of these models. Furthermore, 66.1% of models included at least one multidimensional index as a predictor of overall mortality. Among these, BODE and its variants (BODEX, modified BODE, eBODE) were the most widely used (26.3%), followed by ADO or its updates/variants (22.8%), ABCD GOLD classification systems (15.8%), CODEX and modified CODEX (10.5%), DOSE and COTE (7.0% each).

A summary of the c-statistics achieved by Cox and logistic regression models at each time interval is reported in Table 2A.

The median c-statistic ranged from 0.68 to 0.78, indicating moderate to good performance and stable predictive ability across different time horizons. We also analyzed the distribution of the c-statistic in models including prognostic factors derived from laboratory tests (blood/sputum) or imaging (CT scans) versus models without these factors. We observed modestly higher values in models incorporating these variables at each time point (from +0.03 up to +0.05). Taken together, machine learning (ML) models did not demonstrate superior performance compared with traditional statistical approaches. The small number of models limited any meaningful assessment of the relative performance of specific ML techniques. Full details can be found in Supplementary Table S4.

The quality assessment showed that 87% of models predicting overall mortality were judged to have a high ROB (Supplementary Figure S2). The most critical issues were observed in the “Analysis” domain, and for a specific set of items related to appropriate handling of model overfitting/optimism (30%), handling of missing data (25.8%), numbers of participants (20%), number of candidate predictors (20%), outcome events and events per candidate predictor (20%). Consequently, 40% of studies were excluded from the quantitative synthesis in subgroup analyses due to high or unclear ROB in critical items affecting the summary estimates.

**Figure 5:**
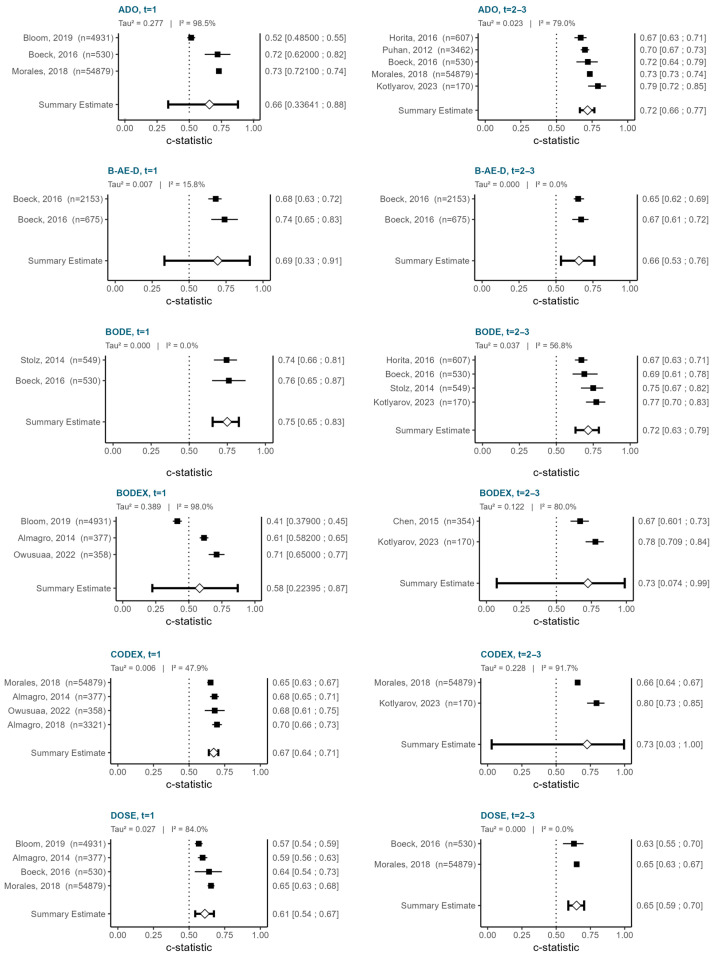
Forest plot depicting results of meta-analysis of c-statistic across models with external validation of multidimensional indices for overall mortality [23,26,29,34,35,38,40,43,52,54,60]. Diamonds represent the pooled estimates with their 95% confidence intervals. Heterogeneity measures: τ^2^ represents the between-study variance; I^2^ is the percentage of variability in the effect sizes that is not caused by sampling error.

The retrieved models were stratified by study characteristics to identify homogeneous groups. Meta-analysis was judged feasible only for traditional statistical models (logistic and Cox regression) validating COPD multidimensional indices [23,26,29,34,35,38,40,43,52,54,60]. Results are shown in Figure 5.

The BODE index achieved the highest pooled c-statistic at 1 year (0.75; 95% CI 0.65–0.83), which was maintained at 2–3 years (0.72; 95% CI 0.63–0.79). However, no more than two or three studies could be included in most of these meta-analyses, and substantial heterogeneity was observed. In some instances, the resulting confidence intervals were extremely wide, limiting any meaningful interpretation of the pooled estimates. No comparable studies were available for meta-analysis of predictive discrimination at 4–5 years. Restricting the analysis to models at low ROB applied in outpatient/primary care recruitment settings [23,29,35,43,54,60] confirmed the superiority of BODE while further narrowing down eligible records for the other indices (Supplementary Figure S5).

### 3.2. Exacerbations

A first set of models was developed for prediction of severe exacerbations (n = 38) [55,56,57,58,59,60,61,62,63,64,66,67,68,69,70,71,72,73,74,75,76,77,78,79,80,81,82]. Of these models, 92.1% were implemented using statistical modelling methods, and only three employed ML approaches (one each of boosting, neural network and random forest). Validation results were reported for 68.4% of models at 1 year, for 18.4% at 2–3 years and for 13.2% at 4–5 years. All ML models were used for short-term predictions at one year. The most frequently used prognostic variables/categories were HPI (94.4%) and spirometry results (77.8%), followed by age (55.6%), physical examination findings (44.4%), sex (38.9%), past medical history (36.1%), smoking status (33.3%), inhaled therapy (22.2%), systemic therapy (16.7%), and blood test results (13.9%). Fifteen models (39.5%) included at least one prognostic score among the predictors, most commonly the ABCD GOLD stages (n = 7, 46.6%). Only three models were applied to hospitalized patients and one in a mixed recruitment setting, leaving a wide predominance (n = 34) from the outpatient/primary care setting. As expected, the rate of a new event requiring hospitalization (while acknowledging differences in outcome adjudication and variability in event reporting) was considerably higher in already hospitalized patients (46.3% vs. 7.5%).

Another 16 models were developed for prediction of moderate or severe exacerbations, none of which employed ML techniques [65,82,83,84,85,86,87]. Validation results were available for nine models (56.2%) at 1 year, for six at 2–3 years, and only for one at 4–5 years. All models incorporated spirometry results, all but one included HPI variables; all other predictors were used in fewer than half of the models. Four models included the ABCD GOLD stages as predictors, while one used the BODEX index.

A summary of the c-statistic distribution across time intervals for models using logistic regression, Cox regression or ML for predictions of severe and severe/moderate exacerbations is presented in Table 2B,C. Both logistic and Cox models for severe exacerbations showed good discriminatory performance at one year (median c 0.77 and 0.74, respectively) but were outperformed by ML models (median c 0.80). Discrimination decreased with longer prediction horizons, declining to a median c of 0.70 at 4–5 years. Fewer models for moderate/severe exacerbations were available, with broadly comparable results.

Significant concerns were identified in the ROB assessment, with only one model both for severe and for moderate/severe exacerbations rated as having a low ROB (Supplementary Figures S3 and S4). This finding was primarily attributable to issues within the “Analysis” domain, notably item 4.3 (exclusion of participants from the analysis), 4.6 (handling of complexities in the data) and 4.4 (handling of missing data).

Given the considerable heterogeneity and the high proportion of studies with elevated ROB, a meta-analysis was deemed inappropriate for either outcome.

## 4. Discussion

This systematic review identified 193 validations of prognostic models developed to predict outcomes related to progression/severity of COPD, primarily targeting all-cause mortality, severe exacerbations, and moderate/severe exacerbations. Other outcomes were explored less frequently and with inconsistent definitions across studies. Most models were based on traditional statistical approaches, predominantly Cox proportional hazards and logistic regression, while ML techniques were used infrequently. From a methodological perspective, Cox regression accounts for time-to-event and censoring, whereas logistic regression and purely classification-based machine learning algorithms do not incorporate follow-up time. This may have important implications when investigating long-term outcomes in prospectively followed cohorts, especially over extended periods of time, where Cox regression and other survival methods are less prone to biased estimates. Across studies, the most consistently included predictors belonged to clinical characteristics of COPD (HPI), spirometry results, age, and physical examination variables, with a substantial proportion also incorporating multidimensional indices such as BODE, ADO, and ABCD GOLD stages. Overall, predictive performance, expressed as the c-statistic, was moderate to good (0.68–0.78) and remained relatively stable across short- to medium-term follow-up horizons. However, the majority of model evaluations were rated as having a high or unclear risk of bias, primarily due to methodological issues in the analysis, thereby limiting their generalizability and potential for clinical implementation. Quantitative syntheses were hampered by heterogeneity in setting, timing and study design. Meta-analysis was feasible only for external validation of a few multidimensional indices in short- to medium-term mortality predictions in primary care settings, showing a superiority of the BODE index. Data were insufficient for long-term outcomes and predictions of exacerbations.

This review is strengthened by its comprehensive search strategy across multiple databases, standardized extraction of model characteristics, and rigorous assessment of applicability and ROB using PROBAST. We also provide detailed categorization of predictors and model types, report performance metrics (c-statistic) across time intervals, and attempt meta-analysis where feasible, offering a nuanced evaluation of COPD prognostic models. Our work integrates and updates prior systematic reviews, which have focused on specific methodologies or individual predicted outcomes [11,14]. Despite a substantial body of literature on prognostic models for COPD, we heavily narrowed down the studies eligible to answer our review question, as those conducted on hospitalized patients and/or patients experiencing an exacerbation and evaluating short-term in-hospital outcomes, such as ICU admissions or the need for mechanical ventilation, were excluded. This exclusion was consistent with the predefined scope and timing criteria of our review, which focused on long-term prognostic outcomes rather than acute events. On the other hand, the recruitment setting of most studies predominantly included patients with moderate to severe COPD. This likely reflects the underdiagnosis of mild disease, as early symptoms can be subtle or nonspecific and spirometric abnormalities may be minimal. Consequently, individuals with mild COPD are less frequently identified in clinical practice or research cohorts, which may limit the generalizability of prognostic models to the full spectrum of disease severity.

Inconsistencies in predictor selection, definition, and measurement contribute substantially to variability across models and complicate direct comparisons of their performance. Overall, the studies followed two broad approaches. The first approach relied on multidimensional indices, often externally validated, which integrate multiple clinical parameters into a composite score. The second approach used predictors that were selected based on data availability within the individual study, whether pre-existing or collected ad hoc. These two strategies reflect differing priorities between leveraging validated tools versus maximizing the use of available data, and both contribute to heterogeneity across models.

The predominance of HPI variables and spirometry as predictors underscores their well-established role in assessing COPD progression. Our findings are consistent with prior systematic reviews [11,14], which identified age, exacerbation history, and lung function measured by spirometry as key predictors in COPD prognostic models. These variables are routinely collected in clinical practice and align with those recommended in COPD assessment and management pathways. Spirometry, for example, is an essential and widely available test, central to guideline-based evaluation. In contrast, other predictors—such as CT-derived imaging features or research-specific biomarkers—are not part of standard COPD assessment and therefore have limited relevance for clinical decision-making. Their use may reduce the applicability of these models in real-world settings, as such tests are neither routinely collected nor widely available, and cannot be applied retrospectively. Nevertheless, it should be noted that only a small number of studies included these types of predictors, limiting their overall impact on the comparability and applicability of the models. In contrast, models developed using health administrative data, which typically include only sociodemographic characteristics, information on type and adherence to therapy, and disease history as reconstructed from healthcare encounters, achieve fair predictive performance and fall shortly behind models incorporating detailed clinical variables. These models offer the advantage of being timely, cost-effective, and applicable to large populations served by healthcare systems covering extensive geographic regions.

The inclusion of multidimensional indices, such as the BODE and ADO scores, reflects an effort to incorporate standardized evaluations of functional status, symptoms, and comorbidities to enhance predictive performance. Their external validation in independent cohorts ensured the use of a small, fixed set of predictors that are routinely collected and consistently defined, making the models more practical, easier to implement, directly applicable across clinical settings, and less prone to bias (Supplementary Table S3). This enabled the quantitative syntheses conducted in this review. Still, the number of studies available for meta-analysis was substantially reduced by the multiplicity of tools, settings and recruited cohorts, follow-up durations, definitions of predicted outcomes and intended applications, which fragmented the evidence base into small and intrinsically incomparable groups of studies. The number was narrowed down further due to widespread methodological issues, even after excluding several PROBAST items from evaluation and thus adopting a more tolerant approach. In the remaining handful of studies, marked heterogeneity in model performance precluded meaningful interpretations of pooled estimates. This ultimately highlights that there are still too few studies dedicated to providing robust external validation of tools that are intended for use in clinical practice.

At the same time, the widespread reliance on multidimensional indices has tended to constrain research to traditional statistical modelling approaches. In contrast, ML approaches were adopted in only a few studies, and even then, primarily to identify the most relevant features from a broad set of potential predictors (e.g., biomarkers, CT image features). Notably, ML approaches remain underutilized despite their potential to model complex interactions among predictors. Consequently, the field appears to lack both robust external validation of existing models and the development of novel, potentially more effective prognostic strategies. As there is growing interest in these approaches for individualized risk prediction, this imbalance is likely to be overturned in the near future.

Most of the published literature has focused on predicting mortality as the most severe outcome. Exacerbations remain relatively understudied, despite being key events in the natural history of COPD. Evaluating the performance of prognostic models for the occurrence of exacerbations was especially hampered by heterogeneity in outcome definitions, such as including exacerbations of varying degrees of severity according to GOLD criteria, considering multiple events, and using combined endpoints. Although these definitions reflect events considered clinically relevant by the study authors, they ultimately hinder comparisons across studies. We suggest that future research should focus on developing prognostic models for exacerbations with robust methodology and standardized definitions of predictors and outcomes, in order to provide valid and clinically actionable tools for healthcare decision-making.

The high ROB in a substantial proportion of studies, particularly related to overfitting, missing data, and participant selection, is consistent with previous reports [11,14] and underscores methodological challenges that compromise the reliability of reported predictive performance. Our methodological assessment revealed the most issues in the “Analysis” domain, including handling of missing data, sample size considerations, mode of validation, and incomplete reporting of performance measures. From a clinical adoption perspective, the lack of external validation prevents any definitive conclusions regarding the generalizability of the reported predictions. Without testing models on independent datasets from different settings, populations, or healthcare systems, it is unclear whether their predictive performance would hold outside the original study. Therefore, these methodological limitations significantly restrict the applicability of the models in other clinical contexts. Additionally, the handling of missing data represents a critical methodological issue. Inadequate management of missing values, such as complete-case analysis without proper assessment or inappropriate imputation, can introduce bias, reduce the precision of model estimates, and limit reliability. This further restricts the applicability of the models in other clinical contexts, as performance observed in the original dataset may not replicate in populations with different patterns of missing data. We also noted that many studies had insufficient sample sizes relative to the number of predictors included in the models. Small sample sizes increase the risk of overfitting, reduce the stability of coefficient estimates, and further limit the generalizability of the findings. Before being applied in a new context, any tool should undergo dedicated validation studies with appropriate design, representative clinical samples, adequate sample sizes, and rigorous methodology to minimize bias. To date, methodological shortcomings in these areas tend to be lower in studies validating indices and higher in studies trying to leverage high-dimensional datasets. In this regard, we found that the few studies employing ML techniques were prone to bias in predictor measurement and appropriateness of analysis (Supplementary Table S5).

Notably, few studies reported assessment of model calibration. Model calibration is crucial, since poor calibration can render predictions misleading [108]. Differences in baseline risks and poor model calibration may well explain why clinically developed models often face difficulties when scaled up to population-level application. Strengthening the evidence base through reproducible methodologies could provide a foundation for innovative approaches, providing models that could ultimately be externally validated and implemented in clinical practice.

## 5. Conclusions

This systematic review was designed to fill a critical gap in the literature by providing the first comprehensive synthesis of both traditional regression-based and ML-based prognostic models across multiple COPD-related outcomes. Previous reviews have been limited in scope, focusing on single outcomes, multivariable regression only, or outdated evidence that does not reflect recent advances in the field. By evaluating model performance qualitatively and quantitatively, we identified predictors and modeling strategies that yield the highest predictive accuracy. Our findings underscore the dominance of regression approaches, the limited adoption of ML, the presence of persistent methodological limitations and the need for more robust, validated models capable of handling complex, multimodal patient data.

While multiple current models demonstrate moderate predictive performance and may support short- and medium-term risk stratification in COPD, particularly for mortality and severe exacerbations, their clinical applicability is restricted by common methodological pitfalls. Even predictions from widely recognized multidimensional indices (BODE, ADO, ABCD GOLD) should be interpreted with caution. Future research should prioritize rigorous external validation in diverse populations, standardized outcome definitions including exacerbation severity and mortality time horizons, transparent and guideline-adherent model development and reporting (e.g., TRIPOD), exploration of ML or hybrid approaches to improve predictive accuracy, integration of multidimensional indices and novel biomarkers to enhance clinical utility.

## Figures and Tables

**Table 1:** Summary characteristics of the cohorts recruited in the studies (n = 96).

Variable	Min	Q1	Median	Q3	Max	N (%) of Cohorts with Reported Information
**Mean age (years)**	58.80	65.50	68.00	71.00	76.30	69 (71.8%)
**% male**	9.55	56.08	70.40	88.38	100.00	70 (72.9%)
**Mean body mass index (kg/m^2^)**	21.65	23.61	25.70	26.95	29.03	51 (53.1%)
**Smoking habit**						
% current smokers (vs. ex/never)	0.00	25.00	32.00	39.00	73.00	49 (51%)
Mean smoking pack-years	18.82	40.68	46.40	51.84	75.60	30 (31.2%)
**Disease severity**						
Mean FEV_1_% predicted	27.00	47.41	49.14	59.90	77.10	44 (54.2%)
% GOLD Stage I	0.00	2.45	8.80	17.00	50.80	27 (28.1%)
% GOLD Stage II	32.00	37.52	42.90	50.20	72.60	26 (27.1%)
% GOLD Stage III	6.40	24.18	32.75	38.60	45.00	26 (27.1%)
% GOLD Stage IV	0.00	5.825	11.75	17.00	22.03	26 (27.1%)
**Treatment**						
% treated with LAMA	4.50	33.60	46.00	56.40	81.10	17 (17.7%)
% treated with LABA	33.50	48.30	56.20	68.05	80.60	19 (19.8%)
% treated with ICS	22.57	47.62	58.69	68.78	87.70	22 (22.9%)

**Table 2:** Distribution of c-statistics performed by models predicting overall mortality (A), severe exacerbations (B), and moderate or severe exacerbations (C) at each time point.

Time (Years)	Model Type	N of Models	Min	Q1	Median	Q3	Max
(**A**) Outcome: overall mortality (n = 85)							
1	Logistic regression	14	0.655	0.723	0.782	0.814	0.832
1	Cox regression	27	0.413	0.652	0.709	0.760	0.926
1	Machine learning	3	0.651	0.652	0.654	0.685	0.716
2–3	Logistic regression	14	0.657	0.670	0.723	0.739	0.789
2–3	Cox regression	25	0.600	0.670	0.709	0.771	0.801
2–3	Machine learning	2	0.650	0.653	0.654	0.655	0.657
4–5	Logistic regression	11	0.620	0.642	0.678	0.695	0.761
4–5	Cox regression	8	0.590	0.692	0.720	0.845	0.920
4–5	Machine learning	0	-	-	-	-	-
(**B**) Outcome: severe exacerbations (n = 38)							
1	Logistic regression	12	0.703	0.723	0.774	0.794	0.861
1	Cox regression	4	0.690	0.728	0.740	0.753	0.790
1	Machine learning	3	0.580	0.688	0.796	0.831	0.866
2–3	Logistic regression	2	0.720	0.735	0.750	0.765	0.780
2–3	Cox regression	3	0.694	0.694	0.694	0.707	0.720
2–3	Machine learning	0	-	-	-	-	-
4–5	Logistic regression	1	0.710	0.710	0.710	0.710	0.710
4–5	Cox regression	4	0.690	0.690	0.695	0.710	0.740
4–5	Machine learning	0	-	-	-	-	-
(**C**) Outcome: moderate or severe exacerbations (n = 16)							
1	Logistic regression	4	0.730	0.748	0.772	0.790	0.790
1	Cox regression	0	-	-	-	-	-
1	Machine learning	0	-	-	-	-	-
2–3	Logistic regression	3	0.660	0.675	0.690	0.735	0.780
2–3	Cox regression	1	0.591	0.591	0.591	0.591	0.591
2–3	Machine learning	0	-	-	-	-	-
4–5	Logistic regression	0	-	-	-	-	-
4–5	Cox regression	1	0.730	0.730	0.730	0.730	0.730
4–5	Machine learning	0	-	-	-	-	-

## Data Availability

The original contributions presented in this study are included in the Supplementary Materials. Further inquiries can be directed to the corresponding author.

## Supplementary Materials

The following supporting information can be downloaded at: https://www.mdpi.com/article/10.3390/jcm14248725/s1, Table S1: PRISMA checklist; Table S2: search strings; Table S3: details of multidimensional prognostic indices [26,35,109,110,111,112,113]; Table S4: data extraction form; Table S5: PROBAST assessment; Table S6: summary characteristics of the study cohorts by setting (outpatient vs. inpatient); Figure S1: methodological workflow of the review; Figure S2: risk of bias assessment of models predicting all-cause mortality (n = 85); Figure S3: risk of bias assessment of models predicting severe exacerbations (n = 38); Figure S4: risk of bias assessment of models predicting moderate or severe exacerbations (n = 16); Figure S5: forest plot depicting results of meta-analysis of c-statistic across models with external validation of multidimensional indices for overall mortality (subgroup).

## Abbreviations

The following abbreviations are used in this manuscript:

ADO	Age, Dyspnea, and airflow Obstruction
B-AE-D	Body mass index, Acute Exacerbations, Dyspnea
BODE	Body mass index, airflow Obstruction, Dyspnea, and Exercise
BODEX	Body mass index, airflow Obstruction, Dyspnea, and EXacerbations
CHARMS	CHecklist for critical Appraisal and data extraction for systematic Reviews of prediction Modelling Studies
CODEX	Comorbidity, airflow Obstruction, Dyspnea, and EXacerbations
COPD	Chronic obstructive pulmonary disease
COTE	COmorbidity TEst
CT	Computed Tomography
DOSE	Dyspnea, airflow Obstruction, Smoking, Exacerbation
FEV_1_	Forced Expiratory Volume in the first second
GOLD	Global Initiative for Chronic Obstructive Lung Disease
HPI	History of present illness
ICS	Inhaled corticosteroids
ICU	Intensive care unit
LABA	Long-acting beta-agonist
LAMA	Long-acting muscarinic antagonist
ML	Machine learning
PRISMA	Preferred Reporting Items for Systematic Reviews and Meta-Analyses
PROBAST	Prediction model Risk Of Bias Assessment Tool
ROB	Risk of bias
