# Supplementary material for: Prognostic Models for Disease Progression and Outcomes in Chronic Obstructive Pulmonary Disease: A Systematic Review and Meta-Analysis
Source: J Clin Med. 2025 Dec 9;14(24):8725. doi: 10.3390/jcm14248725 (PMC12734152; doi:10.3390/jcm14248725)

Prognostic models for disease progression and outcomes in chronic obstructive pulmonary disease: a systematic review and meta-analysis

Deborah Testa[1], Pietro Magnoni[1], Caterina Fanizza[2], Martino Bussa[3], Adele Zanfino[1], Dariush Khaleghi Hashemian[1], Paola Rebora[3,4], Lucia Bisceglia[2], Antonio Giampiero Russo[1] on behalf of the PROPHET-I Study Group

1) *Unità di Epidemiologia, Agenzia di Tutela della Salute (ATS) della Città Metropolitana di Milano, Via Conca del Naviglio 45, 20123 Milan, Italy*
2) *Area Epidemiologia e Care Intelligence, Agenzia Regionale Strategica per la Salute ed il Sociale (AReSS) Pu-glia, 70121 Bari, Italy*
3) *Centro Interdipartimentale Bicocca Bioinformatics Biostatistics and Bioimaging Centre (B4), Dipartimento di Medicina e Chirurgia, Università Milano Bicocca, 20126 Vedano al Lambro, Italy*
4) *Biostatistics and Clinical Epidemiology, Fondazione IRCCS San Gerardo dei Tintori, 20900 Monza, Italy*

Corresponding author:

Pietro Magnoni

Agenzia di Tutela della Salute della Città Metropolitana di Milano – Unità di Epidemiologia

Via Conca del Naviglio 45, 20123 Milano, Italy

E-mail: pmagnoni@ats-milano.it

ORCID 0000-0002-6431-6261

Supplementary Table S1. Compiled PRISMA (2020) checklist for the present systematic review (next page).

PRISMA 2020 Checklist

Section and Topic	Item #	Checklist item	Location where item is reported
**TITLE**			
Title	1	Identify the report as a systematic review.	P 1: LL 3-4
**ABSTRACT**			
Abstract	2	See the PRISMA 2020 for Abstracts checklist.	P 1: LL 17-41
**INTRODUCTION**			
Rationale	3	Describe the rationale for the review in the context of existing knowledge.	PP 2-3: LL 73-94
Objectives	4	Provide an explicit statement of the objective(s) or question(s) the review addresses.	P 3: LL 95-101
**METHODS**			
Eligibility criteria	5	Specify the inclusion and exclusion criteria for the review and how studies were grouped for the syntheses.	PP 3-4: LL 122-141
Information sources	6	Specify all databases, registers, websites, organisations, reference lists and other sources searched or consulted to identify studies. Specify the date when each source was last searched or consulted.	P 3: LL 112-121
Search strategy	7	Present the full search strategies for all databases, registers and websites, including any filters and limits used.	P 3: LL 114-118; Table S1
Selection process	8	Specify the methods used to decide whether a study met the inclusion criteria of the review, including how many reviewers screened each record and each report retrieved, whether they worked independently, and if applicable, details of automation tools used in the process.	PP 3-4: LL 107-108, LL 142-146
Data collection process	9	Specify the methods used to collect data from reports, including how many reviewers collected data from each report, whether they worked independently, any processes for obtaining or confirming data from study investigators, and if applicable, details of automation tools used in the process.	P 4: LL 148-152, LL 162-166
Data items	10a	List and define all outcomes for which data were sought. Specify whether all results that were compatible with each outcome domain in each study were sought (e.g. for all measures, time points, analyses), and if not, the methods used to decide which results to collect.	P 4: LL 155-160
	10b	List and define all other variables for which data were sought (e.g. participant and intervention characteristics, funding sources). Describe any assumptions made about any missing or unclear information.	P 4: LL 152-155, LL 167-177; Table S2
Study risk of bias assessment	11	Specify the methods used to assess risk of bias in the included studies, including details of the tool(s) used, how many reviewers assessed each study and whether they worked independently, and if applicable, details of automation tools used in the process.	PP 4-5: LL 178-197; Table S3
Effect measures	12	Specify for each outcome the effect measure(s) (e.g. risk ratio, mean difference) used in the synthesis or presentation of results.	PP 4-5: LL 158-162, LL 199-200
Synthesis methods	13a	Describe the processes used to decide which studies were eligible for each synthesis (e.g. tabulating the study intervention characteristics and comparing against the planned groups for each synthesis (item #5)).	P 5: LL 200-202

PRISMA 2020 Checklist

Section and Topic	Item #	Checklist item	Location where item is reported
	13b	Describe any methods required to prepare the data for presentation or synthesis, such as handling of missing summary statistics, or data conversions.	NA
	13c	Describe any methods used to tabulate or visually display results of individual studies and syntheses.	P 5: LL 199-202
	13d	Describe any methods used to synthesize results and provide a rationale for the choice(s). If meta-analysis was performed, describe the model(s), method(s) to identify the presence and extent of statistical heterogeneity, and software package(s) used.	P 5: LL 198-207
	13e	Describe any methods used to explore possible causes of heterogeneity among study results (e.g. subgroup analysis, meta-regression).	P 5: LL 202-206
	13f	Describe any sensitivity analyses conducted to assess robustness of the synthesized results.	NA
Reporting bias assessment	14	Describe any methods used to assess risk of bias due to missing results in a synthesis (arising from reporting biases).	NA
Certainty assessment	15	Describe any methods used to assess certainty (or confidence) in the body of evidence for an outcome.	P 5: LL 191-197, LL 202-206
**RESULTS**			
Study selection	16a	Describe the results of the search and selection process, from the number of records identified in the search to the number of studies included in the review, ideally using a flow diagram.	P 5: LL 209-217
	16b	Cite studies that might appear to meet the inclusion criteria, but which were excluded, and explain why they were excluded.	P 5: LL 210-215; Figure S1
Study characteristics	17	Cite each included study and present its characteristics.	PP 5-8: LL 215-271; Figures 1-2; Tables 1, S2
Risk of bias in studies	18	Present assessments of risk of bias for each included study.	P 8: LL 279-284; Table S3
Results of individual studies	19	For all outcomes, present, for each study: (a) summary statistics for each group (where appropriate) and (b) an effect estimate and its precision (e.g. confidence/credible interval), ideally using structured tables or plots.	P 8: LL 272-278; Figure 4; Table S2
Results of syntheses	20a	For each synthesis, briefly summarise the characteristics and risk of bias among contributing studies.	PP 8-11: LL 292-326, LL 347-377; Figures 3, S2-S4;
	20b	Present results of all statistical syntheses conducted. If meta-analysis was done, present for each the summary estimate and its precision (e.g. confidence/credible interval) and measures of statistical heterogeneity. If comparing groups, describe the direction of the effect.	PP 8-11: LL 272-278, LL 313-318, LL 327-345,

Section and Topic	Item #	Checklist item	Location where item is reported
			LL 363-370; Table 2; Figure 4
	20c	Present results of all investigations of possible causes of heterogeneity among study results.	PP 10-11: LL 342-345; Figure S5
	20d	Present results of all sensitivity analyses conducted to assess the robustness of the synthesized results.	NA
Reporting biases	21	Present assessments of risk of bias due to missing results (arising from reporting biases) for each synthesis assessed.	NA
Certainty of evidence	22	Present assessments of certainty (or confidence) in the body of evidence for each outcome assessed.	PP 10-12; LL 338-341, LL 376-397
**DISCUSSION**			
Discussion	23a	Provide a general interpretation of the results in the context of other evidence.	PP 12-13: LL 398-460
	23b	Discuss any limitations of the evidence included in the review.	PP 12-13: LL 431-440, LL 452-457
	23c	Discuss any limitations of the review processes used.	P 12: LL 404-416
	23d	Discuss implications of the results for practice, policy, and future research.	PP 13-14: LL 457-460, LL 470-493
**OTHER INFORMATION**			
Registration and protocol	24a	Provide registration information for the review, including register name and registration number, or state that the review was not registered.	PP 2-3: L 44, LL 109-111
	24b	Indicate where the review protocol can be accessed, or state that a protocol was not prepared.	P 3: LL 109-111
	24c	Describe and explain any amendments to information provided at registration or in the protocol.	NA
Support	25	Describe sources of financial or non-financial support for the review, and the role of the funders or sponsors in the review.	P 14: LL 509-517
Competing interests	26	Declare any competing interests of review authors.	P 14: L 535
Availability of data, code and other materials	27	Report which of the following are publicly available and where they can be found: template data collection forms; data extracted from included studies; data used for all analyses; analytic code; any other materials used in the review.	P 14: LL 520-521; Tables S2-S3

From: Page MJ, McKenzie JE, Bossuyt PM, Boutron I, Hoffmann TC, Mulrow CD, et al. The PRISMA 2020 statement: an updated guideline for reporting systematic reviews. BMJ 2021;372:n71. doi: 10.1136/bmj.n71. This work is licensed under CC BY 4.0. To view a copy of this license, visit https://creativecommons.org/licenses/by/4.0/

Supplementary Table S2. Search strategies.

PUBMED	EMBASE
(pulmonary disease, chronic obstructive OR<br>chronic obstructive OR<br>copd OR<br>coad OR<br>obstructive pulmonary OR<br>obstructive lung OR<br>obstructive airway* OR<br>chronic pulmonary OR<br>chronic lung OR<br>chronic airway* OR<br>emphysema OR<br>bronchitis, chronic)<br>**AND**<br>(prediction OR<br>predictive OR<br>prognostic OR<br>prognosis)<br><br>**AND**<br>(models OR<br>tools OR<br>machine learning OR<br>regression analysis OR<br>regression OR<br>proportional hazards models)<br>**AND**<br>(disease progression OR<br>evolution OR<br>COPD complications OR<br>Chronic obstructive pulmonary disease complications OR<br>exacerbations)	('chronic obstructive lung disease' OR<br>copd OR<br>coad OR<br>'obstructive pulmonary' OR<br>'obstructive lung' OR<br>'obstructive airway*' OR<br>'chronic pulmonary' OR<br>'chronic lung' OR<br>'chronic airway*' OR<br>emphysema OR<br>'bronchitis chronic')<br><br>**AND**<br>('prediction and forecasting'/exp OR<br>prediction OR<br>predictive OR<br>prognostic OR<br>prognosis)<br>**AND**<br>('regression model'/exp OR<br>'cox model' OR<br>models OR<br>tools OR<br>'machine learning'/exp)<br><br>**AND**<br>(progression OR<br>'complication'/exp OR<br>evolution OR<br>'chronic obstructive lung disease complications' OR<br>'copd complications' OR<br>'exacerbations')

Supplementary Table S3 (A-G). Composition, scoring, and references of multidimensional prognostic indices for COPD assessed in the review.

A) Assignment of points (ADO score)	0	1	2	3	4	5
FEV_1 (% predicted)	≥ 65	36–64	≤ 35	—	—	—
Dyspnoea (mMRC)	0–1	2	3	4	—	—
Age (years)	40–49	50–59	60–69	70–79	80–89	≥ 90

Puhan, M. A., Garcia-Aymerich, J., Frey, M., ter Riet, G., Antó, J. M., Agustí, A. G., Gómez, F. P., Rodríguez-Roisín, R., Moons, K. G., Kessels, A. G., & Held, U. Expansion of the prognostic assessment of patients with chronic obstructive pulmonary disease: the updated BODE index and the ADO index. *Lancet* **2009**, *374*, 704–711. https://doi.org/10.1016/S0140-6736(09)61301-5.

B) Assignment of points (BODE score)	0	1	2	3
BMI (kg/m²)	> 21	≤ 21	—	—
FEV_1 (% predicted)	≥ 65	50–64	36–49	≤ 35
Dyspnea (mMRC)	0–1	2	3	4
6-minute walk distance (meters)	≥ 350	250–349	150–249	≤ 149

Celli, B. R., Cote, C. G., Marin, J. M., Casanova, C., Montes de Oca, M., Mendez, R. A., Pinto Plata, V., & Cabral, H. J. The body-mass index, airflow obstruction, dyspnea, and exercise capacity index in chronic obstructive pulmonary disease. *N Engl J Med* **2004**, *350*, 1005–1012. https://doi.org/10.1056/NEJMoa021322.

C) Assignment of points (BODEX score)	0	1	2	3
Body mass index (kg/m²)	> 21	≤ 21	—	—
FEV_1 (% predicted)	≥ 65	50–64	36–49	≤ 35
Number of exacerbations	0	1-2	≥3	

Soler-Cataluña, J. J., Martínez-García, M. A., Sánchez, L. S., Tordera, M. P., & Sánchez, P. R. Severe exacerbations and BODE index: two independent risk factors for death in male COPD patients. *Respir Med* **2009**, *103*, 692–699. https://doi.org/10.1016/j.rmed.2008.12.005 .

D) Assignment of points (DOSE score)	0	1	2	3
Dyspnea (MRC)	0–1	2	3	4
Obstruction (FEV_1 % predicted)	≥50	36–49	21–35	≤20
Smoking (current/past)	Non- smoker	Former smoker	Current smoker	4
Exacerbations (per year)	0–1	2	3	≥4

Jones, R. C., Donaldson, G. C., Chavannes, N. H., Kida, K., Dickson-Spillmann, M., Harding, S., Wedzicha, J. A., Price, D., & Hyland, M. E. Derivation and validation of a composite index of severity in chronic obstructive pulmonary disease: the DOSE Index. *Am J Respir Crit Care Med* **2009**, *180*, 1189–1195. https://doi.org/10.1164/rccm.200902-0271OC.

E) Assignment of points (optimised B-AE-D score)	0	3	6	7	9	10
BMI (kg/m²)	≥ 21		< 21		<18.5	
Number of severe exacerbations in the previous year	0	1		≥2		
Dyspnea (MRC)	0–2		3			4

Boeck, L., Soriano, J. B., Brusse-Keizer, M., Blasi, F., Kostikas, K., Boersma, W., Milenkovic, B., Louis, R., Lacoma, A., Djamin, R., Aerts, J., Torres, A., Rohde, G., Welte, T., Martinez-Camblor, P., Rakic, J., Scherr, A., Koller, M., van der Palen, J., Marin, J. M., … Stolz, D. Prognostic assessment in COPD without lung function: the B-AE-D indices. *Eur Respir J* **2016**, *47*, 1635–1644. https://doi.org/10.1183/13993003.01485-2015.

F) Assignment of points (CODEX score)	0	1	2	3
Charlson comorbidity index	0–4	5-7	≥8	≥4
FEV_1 % predicted	≥65	50-64	36-49	≤35
Dyspnea (MRC)	0–1	2	3	
Severe exacerbations (hospitalizations)	0	1-2	≥ 3	

Almagro, P., Soriano, J. B., Cabrera, F. J., Boixeda, R., Alonso-Ortiz, M. B., Barreiro, B., Diez-Manglano, J., Murio, C., Heredia, J. L., & Working Group on COPD, Spanish Society of Internal Medicine. Short- and medium-term prognosis in patients hospitalized for COPD exacerbation: the CODEX index. *Chest* **2014**, *145*, 972–980. https://doi.org/10.1378/chest.13-1328.

G) Categorization (GOLD ABCD)*		
**CAT score ≥10 or mMRC ≥2**	**≥2 moderate exacerbations or ≥1 hospitalization in the previous year**	**Category**
No	No	**A**
Yes	No	**B**
No	Yes	**C**
Yes	Yes	**D**

*** Used until 2023.**

Vestbo, J., Hurd, S. S., Agustí, A. G., Jones, P. W., Vogelmeier, C., Anzueto, A., Barnes, P. J., Fabbri, L. M., Martinez, F. J., Nishimura, M., Stockley, R. A., Sin, D. D., & Rodriguez-Roisin, R. Global strategy for the diagnosis, management, and prevention of chronic obstructive pulmonary disease: GOLD executive summary. *Am J Respir Crit Care Med* **2013**, *187*, 347–365. https://doi.org/10.1164/rccm.201204-0596PP.

Supplementary Table S6. Summary characteristics of the cohorts recruited in the studies by setting (outpatient vs inpatient).

Setting	Outpatient / primary care (n=75)						Hospitalized (n=15)					
**Variable**	**Min**	**Q1**	**Median**	**Q3**	**Max**	**N (%) of cohorts with reported information**	**Min**	**Q1**	**Median**	**Q3**	**Max**	**N (%) of cohorts with reported information**
**Mean age (years)**	58.8	64.6	67	70.1	75.7	56 (74.7%)	68.8	70.3	71.6	72.5	76.3	10 (66.7%)
**% male**	9.55	55.9	70	88.5	100	57 (76.0%)	51	57.4	72.5	87.4	94	10 (66.7%)
**Mean body mass index (kg/m^2)**	21.6	23.5	26	26.9	29.0	40 (53.3%)	22.8	23.6	25.4	26.9	27.8	9 (60.0%)
**Smoking habit**												
% current smokers (vs ex/never)	0.32	25.9	33.7	42.8	73	39 (52.0%)	21.7	27	31.6	36.6	38.9	6 (40.0%)
Mean smoking pack-years	18.8	40.1	46.4	51.3	75.6	22 (29.3%)	40.9	44.2	48.5	53.6	59.6	6 (40.0%)
**Disease severity**												
Mean FEV_1% predicted	27	48.1	52.5	62.0	77.1	42 (56.0%)	35	41.1	44.1	46.8	54.2	7 (46.7%)
% GOLD Stage I	0	0	8.7	17.8	50.8	25 (33.3%)	9.6	9.6	9.6	9.6	9.6	1 (6.7%)
% GOLD Stage II	32	39.6	43.5	50.7	72.6	24 (32.0%)	37	37	37	37	37	1 (6.7%)
% GOLD Stage III	6.4	23.4	32.8	38.5	45	24 (32.0%)	38.7	38.7	38.7	38.7	38.7	1 (6.7%)
% GOLD Stage IV	0	5.25	9.9	17	22.0	24 (32.0%)	14.8	14.8	14.8	14.8	14.8	1 (6.7%)
**Treatment**												
% treated with LAMA	4.5	34.2	52.2	61.3	81.1	14 (18.7%)	22.0	32.0	42.0	43.8	45.5	3 (20.0%)
% treated with LABA	33.5	50.6	55.8	69.1	80.6	16 (21.3%)	35.6	45.9	56.2	59.6	63.1	3 (20.0%)
% treated with ICS	22.6	47.8	59.9	70.7	87.7	19 (25.3%)	34.6	44.1	53.6	57	60.4	3 (20.0%)

Supplementary Figure S1. Methodologic workflow of this systematic review.

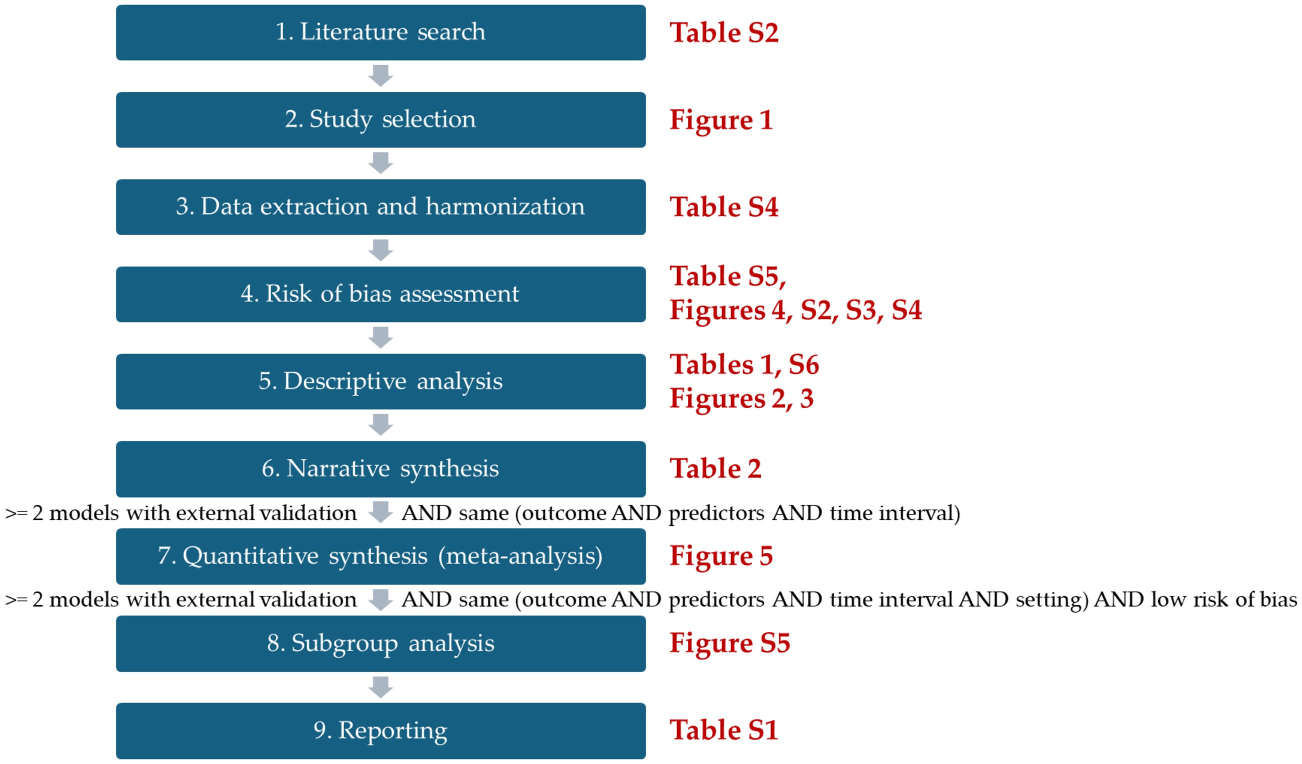

Supplementary Figure S2. Risk of bias assessment (PROBAST) of models predicting all-cause mortality (n=85).

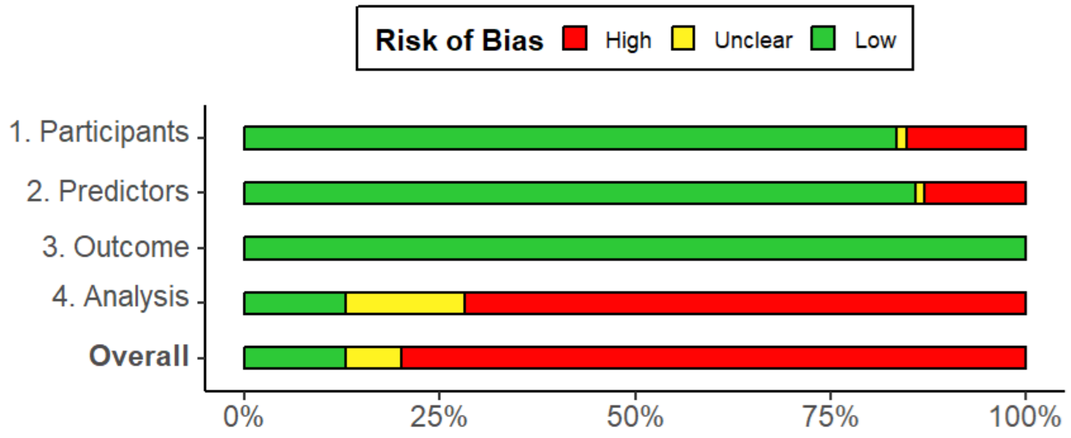

Supplementary Figure S3. Risk of bias assessment (PROBAST) of models predicting severe exacerbations (n=38).

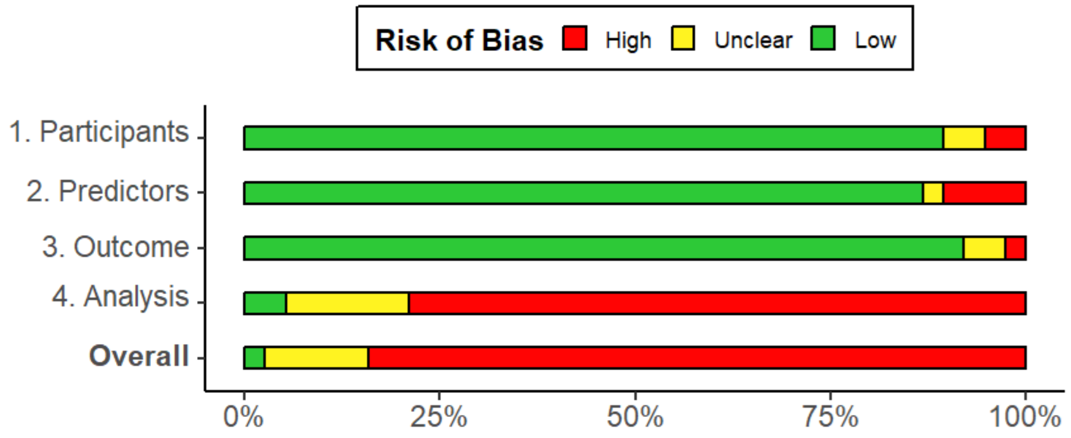

Supplementary Figure S4. Risk of bias assessment (PROBAST) of models predicting moderate or severe exacerbations (n=16).

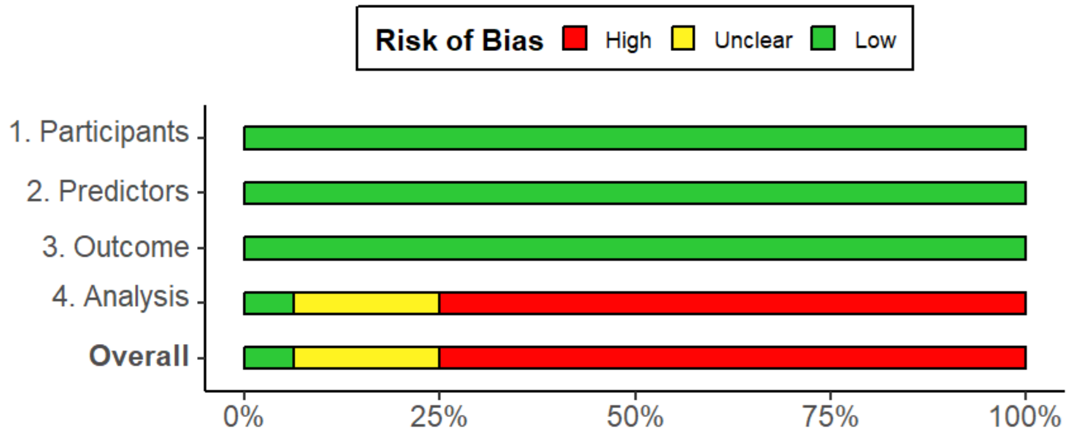

Supplementary Figure S5. Forest plot depicting results of meta-analysis of c-statistic across models with external validation of multidimensional indices for overall mortality. Only models judged at low risk of bias in key PROBAST items and applied in outpatient/primary care recruitment settings are included. Heterogeneity measures: τ^2 represents the between-study variance, I^2 is the percentage of variability in the effect sizes which is not caused by sampling error.

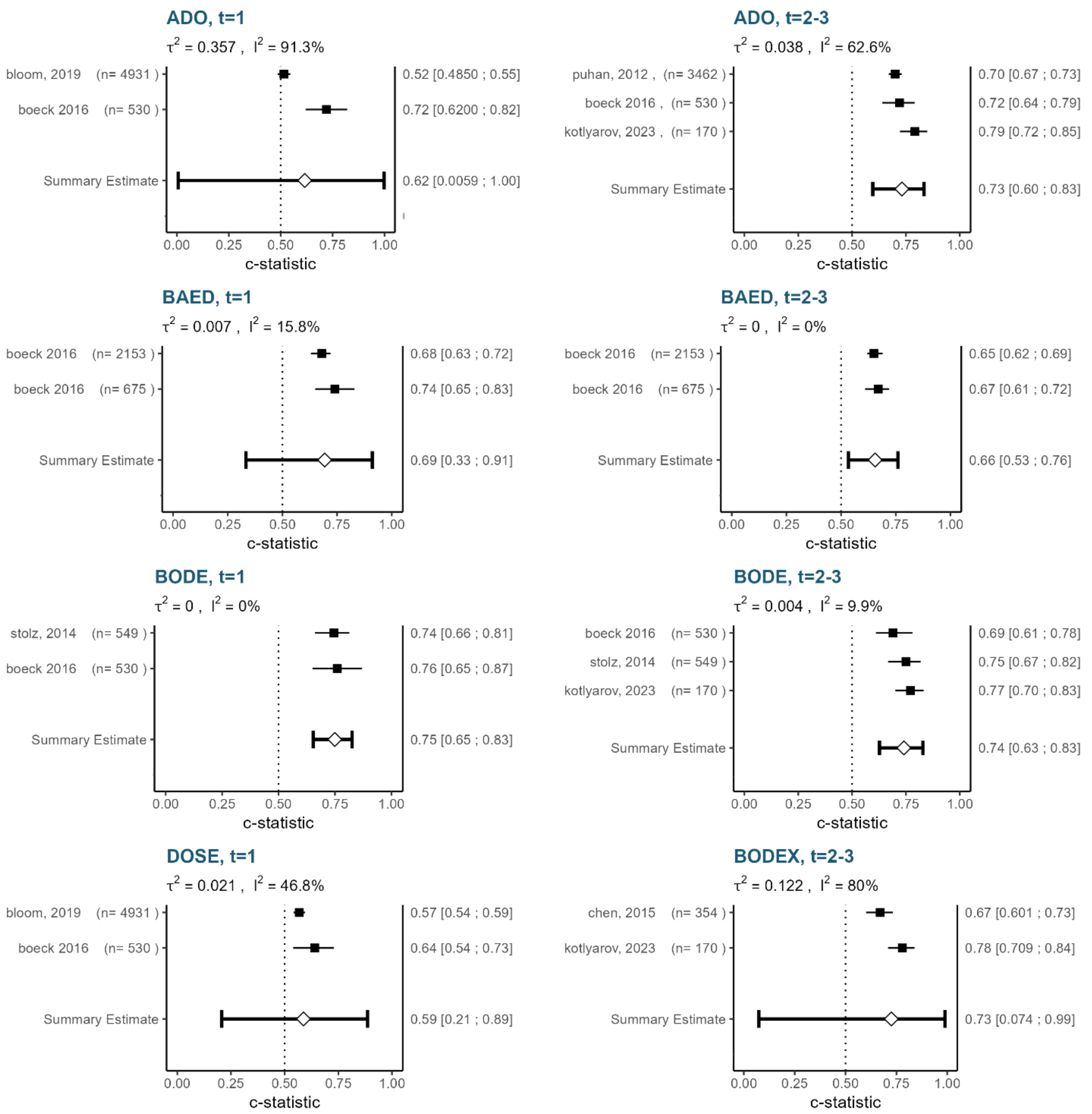

Supplement: Supplementary file 1 [file jcm-14-08725-s001.zip › Supplementary Materials/Supplementary Materials.pdf]
